# Supplementary material for: Bactericidal Effect of Underwater Plasma Treatment on Waste Brine from Kimchi Production Process and the Evaluation of Reusability of Plasma-Treated Waste Brine in Salting Kimchi Cabbage
Source: Foods. 2023 Feb 7;12(4):728. doi: 10.3390/foods12040728 (PMC9956011; doi:10.3390/foods12040728)
Supplement: Supplementary file 1 [file foods-12-00728-s001.zip › foods-2169748-supplementary.pdf]

Appendix A1. Supplementary material

Table S1. 16s rRNA gene sequencing from microorganisms isolated from waste brine

No.	Description	Identities (%)
PCA1	*Bacillus stratosphericus* strain IHB B 6832 16S rRNA gene, partial sequence	99
	Bacillus altitudinis strain IHB B 1045 16S rRNA gene, partial sequence	99
	Bacillus aerophilus strain IHB B 15224 16S rRNA gene, partial sequence	99
PCA2	Uncultured bacterium clone Y27 16S rRNA gene, partial sequence	97
	Paenibacillus sp. O-4c gene for 16S rRNA, partial sequence	97
	Uncultured organism clone ELU0035-T194-S-N1 000090 small subunit rRNA gene, partial sequence	98
PCA3	*Microbacterium testaceum* StLB037 DNA, complete genome	99
	Microbacterium testaceum StLB037 16S rRNA, complete sequence	99
	Microbacterium testaceum strain V12 16S rRNA gene, partial sequence	99
PCA4	*Curtobacterium oceanosedimentum* partial 16S rRNA gene, strain L24	99
	Curtobacterium oceanosedimentum strain JN24 16S rRNA gene, partial sequence	99
	Bacillus subtilis strain TPL16 16S rRNA gene, partial sequence	99
PCA5	*Kocuria palustris* strain M16_2A 16S ribosomal RNA gene, partial sequence	99
	Kocuria sp. BBB3 16S rRNA gene, partial sequence	99
	Kocuria palustris strain MU14/1, complete genome	99
PCA6	*Kocuria palustris* strain IHBB 11063 16S rRNA gene, partial sequence	99
	Kocuria palustris strain MU14/1, complete genome	100
	Kocuria palustris strain 3538 16S rRNA gene, partial sequence	100
PCA7	*Psychrobacter piscatorii* gene for 16S rRNA, partial sequence	99
	Psychrobacter piscatorii strain T-3-2 16S rRNA gene, partial sequence	100
	Gamma *proteobacterium* UMB21A 16S rRNA gene, partial sequence	99
PCA8	*Microbacteriaceae bacterium* SAP771.4 16S rRNA gene, partial sequence	99

	Herbiconiux sp. I10A-02292 16S rRNA gene, partial sequence	99
	Uncultured bacterium clone QQSB17 16S rRNA gene, partial sequence	99
	Microbacterium testaceum strain 343 16S rRNA gene, partial sequence	99
PCA9	*Microbacterium testaceum* strain LM-2 16S rRNA gene, partial sequence	99
	Microbacterium testaceum partial 16S rRNA gene, strain YJM-08	99
	Microbacterium testaceum strain V12 16S rRNA gene, partial sequence	99
PCA10	*Microbacterium testaceum* StLB037 DNA, complete genome	99
	Microbacterium testaceum StLB037 strain StLB037 16S rRNA, complete sequence	99
	Microbacterium oxydans strain IHB B13601 16S rRNA gene, partial sequence	99
PCA11	Uncultured bacterium clone NDB8 16S rRNA gene, partial sequence	99
	Uncultured bacterium gene for 16S rRNA, partial sequence, clone: SC35	99
	Bacterium YC-ZSS-LKJ71 16S rRNA gene, partial sequence	99
PCA12	*Marinilactibactillus psychrotolerans* gene for 16S rRNA	99
	Marinilactibacillus psychrotolerans gene for 16S rRNA, partial sequence, strain: NBRC 100008	99
	Sphingomonas phyllosphaerae strain FA2 16S rRNA gene, partial sequence	99
PCA13	*Sphingomonas phyllosphaerae* strain FA1 16S rRNA gene, partial sequence	99
	Sphingomonas phyllosphaerae 16S rRNA gene, complete sequence	99
	Bacterium YC-ZSS-LKJ71 16S rRNA gene, partial sequence	99
PCA14	*Marinilactibacillus psychrotolerans* gene for 16S rRNA	99
	Marinilactibacillus psychrotolerans gene for 16S rRNA, partial sequence, strain: M13-5	100
PCA15	*Hypocreales* sp. HWJ2(1) 18S rRNA gene, partial sequence; internal transcribed spacer 1, 5.8S rRNA gene, and internal transcribed spacer 2, complete sequence; and 28S rRNA gene, partial sequence	99
	Hypocreales sp. BAB-3884 18S rRNA gene, partial sequence; internal transcribed spacer 1, 5.8S rRNA gene, and internal transcribed spacer 2, complete sequence; and 28S rRNA gene, partial sequence	99

	Uncultured fungus clone CMH100 18S rRNA gene, partial sequence; internal transcribed spacer 1, 5.8S rRNA gene, and internal transcribed spacer 2, complete sequence; and 28S rRNA gene, partial sequence	99
	Sanguibacter inulinus strain ST50 16S rRNA gene, partial sequence	99
PCA16	*Sanguibacter* sp. Everest-gws-55 16S rRNA gene, partial sequence	99
	Sanguibacter suarezii ST50 16S rDNA	99
	Bacillus sp. 6061 16S rRNA gene, partial sequence	99
PCA17	*Bacillus* sp. A5-11 16S rRNA gene, parital sequence	99
	Brevundimonas bullata strain IHBB 9337 16S rRNA gene, parital sequence	99
	Uncultured bacterium clone YT-53 16S rRNA gene, partial sequence	100
PCA18	*Methylobacterium radiotolerans* gene for 16S rRNA, partial sequence, strain: 91a	100
	Methylobacterium sp. 13635G 16S rRNA gene, parital sequence	100
	Arthrobacter oxydans strain IHBB 11072 16S rRNA gene, partial sequence	99
PCA19	*Arthrobacter oxydans* strain 32 16S rRNA gene, parital sequence	99
	Uncultured bacterium clone EMIRGE_OUT_slt2b_203 16S rRNA gene, partial sequence	99
	Bacillus sp. A-3-15 16S rRNA gene, partial sequence	100
PCA20	*Bacillus subtilis* strain H-3 16S rRNA gene, partial sequence	99
	Bacillus megaterium strain Q3, complete genome	99
	Paenibacillus strain DCY03 16S rRNA gene, partial sequence	96
PCA21	*Paenibacillus* sp. NBRC 13579 gene for 16S rRNA, partial sequence	96
	Paenibacillus chinjuensis strain WN9 16S rRNA gene, partial sequence	96
	Bacillus megaterium strain J-65 16S rRNA gene, partial sequence	99
PCA22	*Bacillus subtilis* strain H-3 16S rRNA gene, partial sequence	99
	Bacillus megaterium strain Q3, complete genome	99
	Bacillus megaterium strain J-65 16S rRNA gene, partial sequence	100
PCA23	*Bacillus* sp. No.44 gene for 16S rRNA, partial sequence	99
	Bacillus sp. A-3-15 16S rRNA gene, partial sequence	100
PCA24	Bacterium YC-ZSS-LKJ71 16S rRNA gene, partial sequence	99
	Marinilactibacillus psychrotolerans gene for 16S rRNA	99

	Marinilactibacillus psychrotolerans gene for 16S rRNA, partial sequence for 16S rRNA, partial sequence, strain: NBRC 100008	99
	Uncultured marine bacterium clone Tc-111 16S rRNA gene, partial sequence	100
TSA1	*Methylobacterium fujisawaense* strain S13 16S rRNA gene, partial sequence	100
	Methylobacterium fujisawaense gene for 16S rRNA, partial sequence	100
	Sanguibacter keddieii DSM 10542, complete genome	99
TSA2	*Sanguibacter keddieii* strain DSM 10542 16S rRNA gene, partial sequence	99
	Sanguibacter keddieii strain DSM 10542 16S rRNA gene, complete sequence	99
	Paenibacillus lactis strain VV03 16S rRNA gene, partial sequence	99
TSA3	*Paenibacillus lactis* strain LG2 16S rRNA gene, partial sequence	99
	Paenibacillus lactis strain SCTB114 16S rRNA gene, partial sequence	99
	Uncultured bacterium clone 286 16S rRNA gene, partial sequence	99
TSA4	Uncultured bacterium clone 411 16S rRNA gene, partial sequence	99
	Microbacterium sp. N3 16S rRNA gene, partial sequence	99
	Kocuria palustris strain MU14/1, complete genome	100
TSA5	*Kocuria palustris* strain 3538 16S rRNA gene, partial sequence	100
	Kocuria palustris straiin 3474 16S rRNA gene, partial sequence	100
	Staphylococcus capitis CR01 complete genome	99
TSA6	*Staphylococcus capitis* subsp. capitis strain AYP1020, complete genome	99
	Staphylococcus caprae partial 16S rRNA gene, strain S3	99
	Bacillus circulans strain X3 16S rRNA gene, partial sequence	99
TSA7	*Bacillus circulans* strain G1-4-80 16S rRNA gene, partial sequence	99
	Bacillus circulans strain IHB B 8016 16S rRNA gene, partial sequence	99
	Bacillus sp. 60LGy-1 gene for 16S rRNA, partial sequence	99
TSA8	*Bacillus* sp. N-21 gene for 16S rRNA, partial sequence	99
	Bacillus thermoamylovorans partial 16S rRNA, strain R-7764	99
TSA9	*Psychrobacter piscatorii* gene for 16S rRNA, partial sequence	99
	Psychrobacter piscatorii strain T-3-2 16S rRNA gene, partial sequence	100

	Gamma proteobacterium UMB21A 16S rRNA gene, partial sequence	99
TSA10	*Microbacterium testaceum* strain V12 16S rRNA gene, partial sequence	99
	Microbacterium testaceum StLB037 DNA, complete genome	99
	Microbacterium testaceum StLB037 strain StLB037 16S rRNA, complete sequence	99
TSA11	*Bacillus simplex* strain IHB B 17010 16S rRNA gene, partial sequence	99
	Bacillus simplex strain IHB B 15619 16S rRNA gene, partial sequence	99
	Bacillus simplex strain IHB B 7001 16S rRNA gene, partial sequence	99
TSA12	Uncultured bacterium clone f4h7 16S rRNA gene, partial sequence	99
	Uncultured bacterium clone s2h10a 16S rRNA gene, partial sequence	99
	Uncultured bacterium clone s1s17a 16S rRNA gene, partial sequence	100
TSA13	Bacterium YC-ZSS-LKJ71 16S rRNA gene, partial sequence	99
	Marinilactibacillus psychrotolerans gene for 16S rRNA	99
	Marinilactibacillus psychrotolerans gene for 16S rRNA, partial sequence, strain: NBRC 100008	99
TSA14	*Microbacterium trichothecenolyticum* strain E-5 16S rRNA gene, partial sequence	99
	Uncultured *Microbacterium* sp. clone AtlantisII_a 16S rRNA gene, partial sequence	99
	Microbacterium trichothecenolyticum strain E-1 16S rRNA gene, partial sequence	99
TSA15	*Bacillus infantis* 16S rRNA, complete sequence	99
	Bacillus infantis NRRL B-14911, complete genome	99
	Bacillus sp. CNJ905 PL04 16S rRNA gene, partial sequence	99
TSA16	*Bacillus* sp. S12206 16S rRNA gene, partial sequence	99
	Bacillus simplex strain N25 16S rRNA gene, partial sequence	99
	Bacillus sp. S11208 16S rRNA gene, partial sequence	99
TSA17	*Bacillus* sp. 6061 rRNA gene, partial sequence	99
	Bacillus sp. A5-11 16S rRNA gene, partial sequence	99

	Bacillus sp. TP-Sbiw-C13 16S rRNA gene, partial sequence	99
	Bacterium YC-ZSS-LKJ71 16S rRNA gene, partial sequence	99
TSA18	*Marinilactibacillus psychrotolerans* gene for 16S rRNA	99
	Marinilactibacillus psychrotolerans gene for 16S rRNA, partial sequence, strain: NBRC 100008	99
	Uncultured bacterium clone RS-B13 16S rRNA gene, partial sequence	99
TSA19	Uncultured bacterium clone EDW07B001_137 16S rRNA gene, partial sequence	99
	Uncultured bacterium clone EDW07B001_114 16S rRNA gene, partial sequence	99
	Psychrobacter piscatorii gene for 16S rRNA, partial sequence	99
TSA20	*Psychrobacter piscatorii* strain T-3-2 16S rRNA gene, partial sequence	99
	Gamma proteobacterium UMB21A 16S rRNA gene, partial sequence	99
	Microbacterium trichotecenolyticum strain 3370 16S rRNA gene, partial sequence	100
TSA21	*Microbacterium trichothecenolyticum* strain E-5 16S rRNA gene, partial sequence	99
	Microbacterium trichotecenolyticum strain 332 16S rRNA gene, partial sequence	100
	Bacillus sp. S22906 16S rRNA gene, partial sequence	99
TSA22	*Bacillus subterraneus* strain Lr10/2 16S rRNA gene, partial sequence	99
	Uncultured bacterium clone S_e12 16S rRNA gene, partial sequence	99
	Bacillus subtilis strain H-3 16S rRNA gene, partial sequence	99
TSA23	*Bacillus megaterium* strain Q3, complete genome	99
	Bacillus megaterium gene for 16S rRNA, partial sequence, strain: Beb-46	99
	Marinilactibacillus psychrotolerans gene for 16S rRNA	99
TSA24	Bacterium YC-ZSS-LKJ71 16S rRNA gene, partial sequence	99
	Marinilactibacillus psychrotolerans gene for 16S rRNA, partial sequence, strain: NBRC 100008	99

	Marinilactibacillus psychrotolerans gene for 16S rRNA, partial sequence, strain: NBRC 100008	99
TSA25	*Marinilactibacillus psychrotolerans* gene for 16S rRNA	99
	Marinilactibacillus psychrotolerans strain NBRC 100002 16S rRNA gene, partial sequence	99
	Uncultured bacterium clone L3-B74 small subunit ribosomal RNA gene, partial sequence	99
MA1	*Psychrobacter piscatorii* gene for 16S rRNA, partial sequence	99
	Psychrobacter piscatorii strain T-3-2 16S rRNA gene, partial sequence	100
	Bacillus sp. 18PAM3 16S rRNA gene, partial sequence	99
MA2	*Bacillus licheniformis* strain LZLJ005 16S rRNA gene, partial sequence	99
	Bacillus licheniformis strain CCMMB918 16S rRNA gene, partial sequence	99
	Uncultured *Bacillus* sp. Clone ACH-14S-203 16S rRNA gene, partial sequence	99
MA3	*Bacillus* sp. C-3-8 16S rRNA gene, partial sequence	99
	Bacillus sp. A-3-21 16S rRNA gene, partial sequence	99
	Hypocreales sp. BAB-2884 18S rRNA gene, partial sequence; internal transcribed spacer 1, 5.8S rRNA gene, and internal transcribed spacer 2, complete sequence; and 28S rRNA gene, partial sequence	99
MA4	*Hypocreales* sp. HWJ2(1) 18S rRNA gene, partial sequence; internal transcribed spacer 1, 5.8S rRNA gene, and interanl transcribed spacer 2, complete sequence; and 28S rRNA gene, partial sequence	99
	Uncultured fungus clone CMH100 18S rRNA gene, partial sequence; internal transcribed spacer 1, 5.8S rRNA gene, and internal transcribed spacer 2, complete sequence; and 28S rRNA gene, partial sequence	99
	Bacillus sp. Mixed culture X3-41 16S rRNA gene, partial sequence	98
MA5	*Bacillus oceanisediminis* gene for 16S rRNA, partial sequence	98
	Bacillus circulans strain 5S5 16S rRNA gene, partial sequence	98
MA6	*Psychrobacter piscatorii* gene for 16S rRNA, partial sequence	99
	Psychrobacter piscatorii strain T-3-2 16S rRNA gene, partial sequence	100

	Gamma proteobacterium UMB21A 16S rRNA gene, partial sequence	99
	Psychrobacter piscatorii gene for 16S rRNA, partial sequence	99
MA7	*Psychrobacter piscatorii* strain T-3-2 16S rRNA gene, partial sequence	100
	Gamma proteobacterium UMB21A 16S rRNA gene, partial sequence	99
	Plantibacter sp. NJ-81 16S rRNA gene, strain NJ-81	100
MA8	*Plantibacter* sp. S51 partial 16S rRNA gene, strain S51	99
	Uncultured *Plantibacter* sp. Gene for 16S rRNA, partial sequence, clone:1X72	99
	Psychrobacter piscatorii gene for 16S rRNA, partial sequence	99
MA9	*Psychrobacter piscatorii* strain T-3-2 16S rRNA gene, partial sequence	99
	Gamma proteobacterium UMB21A 16S rRNA gene, partial sequence	99
	Microbacterium testaceum strain LM-2 16S rRNA gene, partial sequence	99
MA10	*Microbacterium testaceum* partial 16S rRNA gene, strain YJM-08	99
	Microbacterium sp. Acj 118 gene for 16S rRNA, partial sequence	99
	Plectosphaerella cucumerina isolate Ecu215 18S rRNA gene, partial sequence; internal transcribed spacer 1, 5.8S rRNA gene, and internal transcribed spacer 2, complete sequence; and 28S rRNA gene, partial sequence	99
MA11	*Plectosphaerella cucumerina* isolate Ecu212a 18S rRNA gene, partial sequence; internal transcribed spacer 1, 5.8S rRNA gene, and internal transcribed spacer 2, complete sequence; and 28S rRNA gene, partial sequence	99
	Trichurus spiralis strain HSAUP052611 18S rRNA gene, partial sequence; internal transcribed spacer 1, 5.8S rRNA gene, and internal transcribed spacer 2, complete sequence; and 28S rRNA gene, partial sequence	99
	Psychrobacter piscatorii gene for 16S rRNA, partial sequence	99
MA12	*Psychrobacter piscatorii* strain T-3-2 16S rRNA gene, partial sequence	100
	Gamma proteobacterium UMB21A 16S rRNA gene, partial sequence	99
	Microbacterium testaceum strain LM-2 16S rRNA gene, partial sequence	99
MA13	*Microbacterium* sp. Acj 118 gene for 16S rRNA, partial sequence	99
	Mcirobacterium testaceum strain 343 16S rRNA gene, partial sequence	99

	Kocuria palustris strain MU14/1, complete genome	100
MA14	*Kocuria palustris* strain IHBB 11063 16S rRNA gene, partial sequence	100
	Kocuria palustris strain 3538 16S rRNA gene, partial sequence	100
	Bacillus sp. EC2 16S rRNA gene, partial sequence	99
MA15	*Bacillus* sp. IARI-HHS2-45 16S rRNA gene, partial sequence	99
	Bacillus sp. 3549BRRJ 16S rRNA gene, partial sequence	99
	Bacillus simplex strain IHB B 17010 16S rRNA gene, partial sequence	99
MA16	*Bacillus* sp. S12206 16S rRNA gene, partial sequence	99
	Bacillus sp. S11208 16S rRNA gene, partial sequence	99
	Rhodococcus sp. FXJ8.139 16S rRNA gene, partial sequence	99
MA17	*Rhodococcus* sp. 7B-577 16S rRNA gene, partial sequence	99
	Rhodococcus sp. K4-07B 16S rRNA gene, partial sequence	99
	Kocuria palustris strain MU14/1, complete genome	100
MA18	*Kocuria palustris* strain IHBB 11063 16S rRNA gene, partial sequence	100
	Kocuria palustris strain 3538 16S rRNA gene, partial sequence	100
	Rhodococcus boritolerans gene for 16S rRNA, partial sequence, strain: BTM-1c	99
MA19	Uncultured bacterium clone Md-129 16S rRNA gene, partial sequence	99
	Uncultured bacterium clone Md-125 16S rRNA gene, partial sequence	99
	Aeromicrobium sp. 1/4_C7/16_31 16S rRNA gene, partial sequence	99
MA20	*Aeromicrobium* alkaliterrae strain KSL-107 16S rRNA gene, partial sequence	99
	Aeromicrobium sp. VO40-3 16S rRNA gene, partial sequence	99
	Uncultured bacterium clone EDW07B001_137 16S rRNA gene, partial sequence	99
MA21	Uncultured bacterium clone EDW07B001_114 16S rRNA gene, partial sequence	99
	Uncultured bacterium clone EDW07B001_58 16S rRNA gene, partial sequence	99

	Alternaria alternata strain 16/10 18S rRNA gene, partial sequence; internal transcribed spacer 1, 5.8S rRNA gene, and internal transcribed spacer 2, complete sequence; and 28S rRNA gene, partial sequence	94
MA22	*Ulocladium chartarum* strain E-000535862 18S rRNA gene, partial sequence; internal transcribed spacer 1, 5.8S rRNA gene, and internal transcribed spacer 2, complete sequence; and 28S rRNA gene, partial sequence	99
	Ulocladium sp. 10a2-1-s 18S rRNA gene, partial sequence; internal transcribed spacer 1, 5.8S rRNA gene, and internal transcribed spacer 2, complete sequence; and 28S rRNA gene, partial sequence	99
	Bacillus sp. A-3-8 16S rRNA gene, partial sequence	100
YPD1	*Bacillus* sp. A-3-7B 16S rRNA gene, partial sequence	100
	Bacillus sp. A-2-17 16S rRNA gene, partial sequence	100
	Verticillium dahliae strain F724 18S rRNA gene, partial sequence; internal transcribed spacer 1, 5.8S rRNA gene, and internal transcribed spacer 2, complete sequence; and 28S rRNA gene, partial sequence	100
YPD2	Uncultured Verticillium clone Reps_1522 18S rRNA gene, internal transcribed spacer 1, 5.8S rRNA gene, internal transcribed spacer 2, and 28S rRNA gene, complete sequence	99
	Gibellulopsis nigrescens strain M331 18S rRNA gene, partial seequence; internal transcribed spacer 1, 5.8S rRNA gene, and internal transcribed spacer 2, complete sequence; and 28S rRNA gene, partial sequence	99
	Pantoea agglomerans strain NSF 16S rRNA gene, partial sequence	100
YPD3	*Curtobacterium plantarum* strain S4 16S rRNA gene, partial sequence	100
	Curtobacterium plantarum strain S2 16S rRNA gene, partial sequence	100
	Curtobacterium oceanosedimentum partial 16S rRNA gene, strain L24	100
YPD4	*Curtobacterium oceanosedimentum* strain JN24 16S rRNA gene, partial sequence	100
	Bacillus subtilis strain TPL16 16S rRNA gene, partial sequence	100
YPD5	Uncultured bacterium clone f4h7 16S rRNA gene, partial sequence	99

	Uncultured bacterium clone s2h10a 16S rRNA gene, partial sequence	99
	Serratia marcescens isolate CECRIbio 01 16S rRNA gene, complete sequence	99
	Bacillus sp. A-3-15 16S rRNA gene, partial sequence	100
YPD6	*Bacillus subtilis* strain H-3 16S rRNA gene, partial sequence	100
	Bacillus megaterium strain Q3, complete genome	100
	Curtobacterium sp. W2.10-183 16S rRNA gene, complete sequence	100
YPD7	*Curtobacterium* sp. D-17 16S rRNA gene, partial sequence	100
	Curtobacterium sp. SAp758.3 16S rRNA gene, partial sequence	100
	Rathayibacter caricis strain VKM Ac-1799 16S rRNA gene, partial sequence	99
YPD8	*Rathayibacter tritici* strain IHBB 9484 16S rRNA gene, partial sequence	99
	Rathayibacter tritici strain DSM 7486 16S rRNA gene, partial sequence	99
	Bacterium RSB-1 gene for 16S rRNA, partial sequence	99
YPD9	Uncultured bacterium clone 286 16S rRNA gene, partial sequence	99
	Uncultured bacterium clone 411 16S rRNA gene, partial sequence	99
	Pantoea agglomerans strain WSB 16S rRNA gene, partial sequence	100
YPD10	*Pantoea agglomerans* strain SSF 16S rRNA gene, partial sequence	100
	Pantoea agglomerans strain QSA 16S rRNA gene, partial sequence	100
	Microbacterium testaceum strain V12 16S rRNA gene, partial sequence	99
YPD11	*Microbacterium testaceum* StLB037 DNA, complete genome	99
	Microbacterium testaceum StLB037 strain StLB037 16S rRNA, complete sequence	99
	Lactococcus lactis subsp. Cremoris gene for 16S rRNA, partial sequence, strain: Ni793	99
YPD12	*Lactobacillus curvatus* gene for 16S rRNA, partial sequence, strain: JCM 1096	99
	Uncultured bacterium clone MY 40 16S rRNA gene, partial sequence	99
	Microbacterium testaceum partial 16S rRNA gene, strain YJM-08	99
YPD13	*Microbacterium* sp. Acj 118 gene for 16S rRNA, partial sequence	99
	Microbacterium testaceum strain LM-2 16S rRNA gene, partial sequence	99

	Bacillus siamensis strain IHB B 15652 16S rRNA gene, partial sequence	99
YPD14	*Bacillus amyloliquefaciens* strain SWM1 16S rRNA gene, partial sequence	99
	Bacillus siamensis strain IHB B 14741 16S rRNA gene, partial sequence	99
	Bacillus methylotrophicus strain YJ11-1-4, complete genome	100
YPD15	*Bacillus amyloliquefaciens* strain L-S60, complete genome	100
	Bacillus amyloliquefaciens strain L-H15, complete genome	100

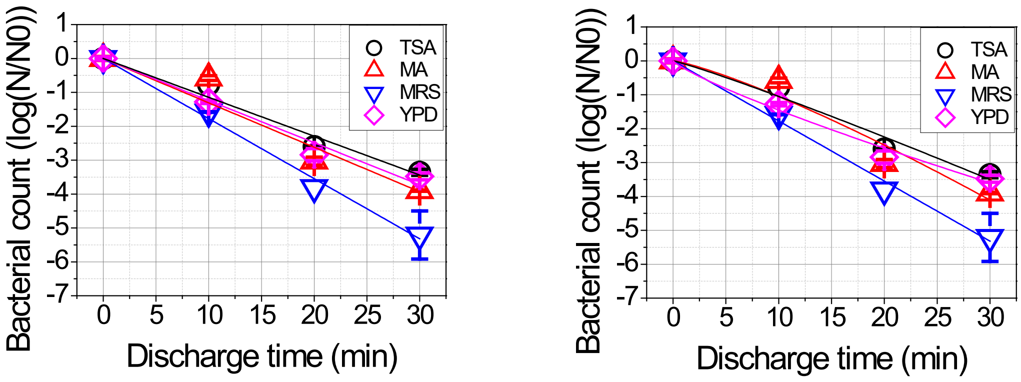

(**a**) (**b**)

Figure S1. The Log-linear and Weibull model fitted to the inactivation rate of microorganisms cultured by 4 different agars. (**a**) the Log-linear model, and (**b**) the Weibull model
